# Preparation and Characterization of Nonwoven Fibrous Biocomposites for Footwear Components

**DOI:** 10.3390/polym12123016

**Published:** 2020-12-16

**Authors:** Fahanwi Asabuwa Ngwabebhoh, Nabanita Saha, Hau Trung Nguyen, Urška Vrabič Brodnjak, Tomas Saha, Anežka Lengalova, Petr Saha

**Affiliations:** 1 Footwear Research Centre, University Institute, Tomas Bata University in Zlin, Nad Ovcirnou IV, 3685 Zlin, Czech Republic; tsaha@utb.cz (T.S.); saha@utb.cz (P.S.); 2 Centre of Polymer Systems, University Institute, Tomas Bata University in Zlin, Tr. T. Bati 5678, 76001 Zlin, Czech Republic; hnguyen@utb.cz (H.T.N.); lengalova@utb.cz (A.L.); 3 Faculty of Technology, Tomas Bata University in Zlin, Vavrečkova 275, 76001 Zlin, Czech Republic; 4 Department of Textiles Graphic Arts and Design, Faculty of Natural Sciences and Engineering, The University of Ljubljana, Snežniška 5, SI 1000 Ljubljana, Slovenia; urska.vrabic@ntf.uni-lj.si; 5 Faculty of Humanities, Tomas Bata University in Zlin, Stefanikova, 5670 Zlin, Czech Republic

**Keywords:** biomass, biocomposite, leather alternative, optimization, eco-friendly material

## Abstract

Chromium-tanned leathers used in the manufacture of footwear and leather goods pose an environmental problem because they contain harmful chemicals and are very difficult to recycle. A solution to this problem can be composite materials from tree leaves, fruit residues and other fibrous agricultural products, which can replace chromium-tanned leather. The present study describes the preparation of biocomposite leather-like materials from microbial cellulose and maple leave fibers as bio-fillers. The formulation was optimized by design of experiment and the prepared biocomposites characterized by tensile test, FTIR, DMA, SEM, adhesion test, volume porosity, water absorptivity, surface wettability and shape stability. From the viewpoint of future use in the footwear industry, results obtained showed that the optimized material was considerably flexible with tensile strength of 2.13 ± 0.29 MPa, elastic modulus of 76.93 ± 1.63 MPa and porosity of 1570 ± 146 mL/min. In addition, the material depicted good shape stability and surface adhesive properties. The results indicate that a suitable treatment of biomass offers a way to prepare exploitable nonwoven fibrous composites for the footwear industry without further burdening the environment.

## 1. Introduction

The term ‘’sustainable development” is gradually becoming an inevitable factor in the footwear industry since conventional leather materials cause environmental hazards due to the use of harmful substances such as chromium, chemical-based adhesives and synthetic rubbers [1,2,3,4]. In recent years, most shoe components are majorly composed of synthetic leather, produced from polyurethane (PU) and polyvinyl chloride (PVC) that requires at least 50 years to fully decompose in the absence of recyclable techniques such as mechanical regrinding and chemical recycling (pyrolysis, hydrogenation etc.) [5]. As such, the increasing environmental and societal effects of these materials have pushed research endeavors towards the development of eco-friendly products in order to resonate the concept of sustainability [6,7]. So far, the footwear industry has been diligently in the incorporation of environmentally friendly materials to their new designs, which is gradually decreasing the carbon footprint cause by PU and PVC based leather products [8].

The fabrication of biocomposites using agro-waste biomass as bio-fillers has tremendously attracted wide application in different material areas considering it improves mechanical property, it is economic and environmentally friendly. Over the years, this technique has proven to be applicable in the automobile, aerospace and construction industries and to a lesser extent the textile and footwear sector. Following studies in the textile and footwear sector, these materials have served as ethical alternative to conventional leather while embracing our fickle fashion desires [9,10]. For example, wastes generated from agricultural biomass, food, textile, and paper pulp processes have proven high end-use potential applications in the production of biodegradable and flexible composites as well as recyclable textile and footwear components [11,12,13,14,15]. Several studies on the use of agricultural biomass from corn and rice husks, banana leaves, sugarcane straw, pineapple leaves and soybean straw have been explored as bio-filler sources and reported as potential natural fiber reinforcers for the production of biocomposites [16,17,18]. These bio-fillers shown a great deal of interest in the development of viable green technologies aimed at the enhancement of composite materials.

In view of global environmental issues and limited fiber sources with good mechanical properties of natural fibers, scientists worldwide have shown increasing interest on exploiting the full potentials of fibers. This has led to several attempts to prepare and evaluate different natural fiber sources. Bacterial cellulose (BC) also known as microbial cellulose, which complements the hydrophobicity of different synthetic polymers with its unique properties such as low cost, biocompatible and mechanical properties makes it a vital additive for composite materials [19,20]. Compared to cellulose extracted naturally from wood pulp and algae, BC has thinner microfibrillar structure leading to better mechanical properties such as tensile strength and Young’s Modulus [21,22,23]. BC as a potential fiber source has found suitable application in the development and design of textile and footwear materials. A study by Shah et al. reported the preparation of microbial cellulose based textiles and the results showed that the prepared fabrics possessed remarkable properties such as good flexibility, biocompatibility and biodegradability, along with unique mechanical strength [24]. Another study by Garcia et al. [25] investigated the properties of microbial cellulose as a leather substitute. The authors described the tactile characteristics of microbial cellulose based materials and their properties as soft and stretchable, similar to those of fine finished leather. Recently, Nam et al. investigated and reported the structural properties of a multi-layered microbial cellulose material supported by denim and hemp fibers. They described the material as able to protect consumers’ feet in thermal equilibrium comparable to commercial calf and pig skin [5]. In addition, several start-ups companies such as Vegea, ScobyTec, or others are coming up with promising industrial production of eco-friendly footwear leather-like materials, such as Malai biocomposite or Pinatex, by converting agro-biomass from fruits and plant leaves as raw natural fiber sources [26,27].

Agro-biomass as bio-fillers has been widely applied in the preparation of composites and has proven to improve material performance in terms of biodegradability, cost-effectiveness and environmental friendliness. Herein, we describe the production of new nonwoven fibrous biocomposites following the homogenous blending of cellulose fibers from bacteria and plant tree leaves as potential bio-fillers with polycaprolactone (PCL), polylactic acid (PLA) and polyvinyl alcohol (PVA) as the polymer matrix. To this aim, Design of Experiment (DoE) was explored as a suitable method to evaluate the formulation of the biocomposite materials [28,29,30,31]. The mixture composition of the prepared biocomposites was optimized. Statistical validation by analysis of variance (ANOVA) was also employed to ascertain the significance of the design model used.

Overall, emphasis of the present study was placed on biocomposite material development and characterization from the perspective of future use in the footwear industry for the production of shoe components. To the best of our knowledge, this is one of the first reports on such formulated biocomposite materials to be used as a footwear leather substitute.

## 2. Materials and Methods

### 2.1. Materials

Microbial cellulose was cultured using the bacterial strain *Komagataeibacter xylinus* (*Gluconacetobacter xylinus*, CCM3611) in the Microbiology Laboratory of the Centre of Polymer Systems, Tomas Bata University in Zlin (TBU), Czech Republic. Maple tree leaves as an additional source of natural fiber bio-filler was collected from public parks near TBU. Polycaprolactone (PCL, M_n_ = 80,000 g/mol), sodium hydroxide (NaOH), and dichloromethane (DCM) were supplied by Sigma-Aldrich (Darmstadt, Germany). Polylactic acid (PLA-4043D) was purchased from NatureWorks LLC (Ingeo^®^, Minnetonka, MN, USA). Glycerol (GL) was supplied by Lachner Co. Ltd. (Neratovice, Czech Republic) and commercial grade polyvinyl alcohol fiber (PVA grade: 55–88) was supplied by Kuraray Poval^™^ (Kamisu, Japan). All reagents were used without further purification. The prepared bio-leather surface morphology was compared with commercial PU leather supplied by Bata shoe factory, Zlin, Czech Republic.

### 2.2. Production of Bio-fillers

The synthesis of microbial cellulose, also known as bacterial cellulose (BC), was performed using the bacteria strain *Komagataeibacter xylinus* in standard HS medium as described previously [32]. In brief, 100 mL culture glass bottles containing predetermined amounts of HS medium inoculum, kombucha biomass (black tea and waste apple juice extract) and bacteria culture mixture were incubated at 30 °C for 15 days. The produced BC membranes were harvested, alkali treated, washed severally to neutral pH and stored in the refrigerator (≈ 4 °C) for further use.

The extraction of maple leave (ML) pulp was carried out via an alkali treatment process as previously reported [33] with slight modifications. Shredded maple leaves were subjected to drying at 45 °C overnight before the treatment procedure itself. Initially, 50 g of the shredded maple leaves was transferred into a 500 mL glass vessel containing 200 mL 8–12% NaOH solution. The mixture was boiled in the temperature range 150 to 200 °C for 2 h followed by cooling down to room temperature. Subsequently, the sample was washed with tap water to pH 7–9. The mixture was then vigorously blended in distilled water at 30,000 rpm for 1 min using a NutriBullet^®^ blender. The ML mixture was centrifuged at 10,000 rpm for 10 min on a Sorvall Lynx 4000 super speed centrifuge (Thermo Scientific™, Waltham, MA USA). Finally, the precipitate was collected, dried at 50 °C overnight to constant weight and stored to be used later.

### 2.3. Preparation of Biocomposite Leather

The biocomposite leathers were prepared by blending different amounts of treated BC and ML with biodegradable polymer matrix of PLA, PCL and PVA. The different component contents were calculated in terms of weight percent (% w/w).

Initially, 25% GL, 25% ML and 10% PVA were homogenously mixed using a micro ball mill (Lab Wizz 320, Laarmann Group, Limburg, The Netherlands) for 2 min at a frequency rate of 25 Hz to produce finely mixed paste. This was compression molded with a prepared film consisting 20% *w/v* PCL (dissolved in DCM) to produce multilayer biocomposite sheets designated as S1.

Similarly, the second sample (S2) was prepared by thoroughly mixing 10% ML, 30% BC and 10% PVA fibers, which was followed by blending with 20% PCL (dissolved in DCM).

Another formulation (S3) was prepared by homogenously blending 30% BC, 10% ML and 10% PVA fibers, which was then mixed with 20% PCL:5% PLA solution. Based on pre-trial experiments, an optimal ratio of 4:1 PCL:PLA was maintained for the preparation of S3.

All homogenously prepared samples (S1, S2 and S3) were molded under compression using a hydraulic press of 5 to 20 kN force with stainless mold frames of 2 mm thick. The formed sheets were cured at 120 °C for 10 min and ambient-air dried for 1–5 days to reach constant weight. The samples’ thickness was measured with an electronic digital micrometer (accuracy 0.001 mm) after the drying process with average values recorded over five different points.

### 2.4. Material Characterization Techniques

#### 2.4.1. Fourier Transform Infrared Spectroscopy (FT-IR)

FT-IR spectra were acquired using a Nicolet iS5 (Thermo Scientific, Waltham, MA, USA) spectrometer in ATR module, to examine the chemical nature of the biocomposites. The spectra were obtained with an accumulation of 64 scans and a resolution of 4 cm^–1^.

#### 2.4.2. Mechanical Tests

The tensile tests were conducted using Instron 5567 (Instron, Norwood, MA, USA) with a static load of 5 kg and a crosshead speed of 10 mm/min at room temperature (25 °C). The samples were cut in the dimension of 50 × 15 mm and the tensile, tear strength and elongation at break tests was measured following the ASTM D882 standard. For each sample, five specimens were evaluated and the average values recorded.

Studies on the thermal behavior of pre-conditioned biocomposite samples was carried out by observing the changes in the storage (E′) and loss (E″) moduli as well as the damping factor (tan delta) using a dynamic mechanical analyzer (DMA Q-800, TA instruments, New Castle, DE, USA). Following pre-trial analysis, the tension deformation mode via sinusoidal oscillatory was applied in the temperature range from –25 °C to 150 °C at a heating rate of 3 °C/min by small strain amplitude (10^–6^) oscillatory tests (linear viscoelasticity) under a frequency sweep mode between 1 and 10 Hz. For this measurement, the specimen’s dimensions were 50 mm in length and 7 mm in width. Prior to the test, the samples were cooled from ambient temperature to –25 °C with liquid nitrogen and then gradually heated to 150 °C.

#### 2.4.3. Morphological Analysis

The morphological properties of the prepared biocomposites were studied and compared to commercial synthetic polyurethane leather using a scanning electron microscope (SEM, FEI™, Brno, Czech Republic) at an accelerating voltage of 5 kV. Prior to analysis, the samples were gold sputter-coated for better surface conductivity using a JEOL JFC 1300 Auto Fine coater (Tokyo, Japan). The transverse section micrographs of the materials were captured at 200× and 1000× magnification.

#### 2.4.4. Surface Wettability Measurements

Wettability of the samples was evaluated by contact angle measurement. The sessile drop technique was applied for this purpose using an Advex Instrument (Brno, Czech Republic) equipped with a CCD camera. Distilled water was used as the testing liquid and the measurements were performed at 25 °C and 50% relative humidity. The droplets volume was set to 10 μL for all experiments and photographic images taken after the deposition of every drop on the biocomposite surface. The measured contact angle value was an average of 10 replicate measurements.

#### 2.4.5. Adhesion Tests

The adhesion test of the prepared biocomposites was performed via the pull-off method (as outlined in ASTM D 4541 and similarly in BS EN ISO 4624), which involved the gluing of a test dolly of 20 mm diameter to the sample surface. The sample’s dimensions were 50 × 50 mm. The experiment was conducted using two components of Araldite epoxy glue parts A and B, mixed in equal amounts (50:50) measured by weight. The time of adhesive cure for all samples was 24 h at room temperature. Subsequently, pulling of the dolly off the samples was carried out using an Elcometer 510 adhesion tester (Elcometer, Manchester, UK) by applying the force perpendicular to the surface of the sample. The pulling force at which the separation occurred was then recorded as the adhesion strength of the material. Readings were performed in triplicate and the mean value was calculated. Finally, the type of failure was determined.

#### 2.4.6. Consumer Properties Measurements

To identify the properties of the prepared biocomposite from the viewpoint of future consumers, water absorption, shape stability, and porosity (expressed in terms of air flow rate) were analyzed.

The static water absorption (SWA) test was carried out by BASF standards of leather technology [34]. Samples of dimensions 15 × 20 mm were completely immersed in water, collected in periodic intervals (1 h, 2 h and 24 h), blotted with tissue paper to remove excess water and weighed. Readings were collected in triplicate and the water absorption was calculated as the percentage increase in the samples’ weight after the given time.

The static water absorption percentage was calculated following Equation (1).

(1) $$\mathrm{SWA}\%=\;\frac{W_{O}}{W_{A}}\;\times 100\;$$

where, W_O_ is the weight of the biocomposite material before immersion (g) and W_A_ is the weight of material after absorption at time, *t*.

The apparent density of the prepared materials was determined according to ASTM D2346 and BASF standards for leather-like materials [34]. Prior to analysis, the test samples were cut in round disk shapes with an average diameter of 20 mm and conditioned for 48 h at 25 °C and 50% relative humidity. Subsequently, the samples were weighed and their thickness measured in four quadrants of the specimen disk, equidistant from the rim and center. All values were collected in triplicate and averaged. The apparent densities were then calculated as the mass per unit volume.

Dimensional stability of a material is a measure of the linear change of the dimension resulting from exposure to temperature. This property indicates uniformity with regard to internal stress introduced during processing. In this study, the test was performed according to ASTM D1204 standard. Firstly, the biocomposite materials were cut into 30 × 20 mm samples on which reference marks were drawn. The specimens were then placed in an oven for a specified time and the temperatures varied (50, 100 and 150 °C). After pre-determined time, the samples were then removed from the oven and conditioned again at room temperature. The distances between the reference marks were re-measured and the average values recorded. The dimensional stability of the material was calculated as the percentage change of the specimen length after heating using Equation (2).

(2) $$\mathrm{Percent}\;\mathrm{change}\;\left(\%\right)\;=\frac{L_{F\;}-L_{O}}{L_{O}}\;\times 100\;$$

where, L_F_ is the final length (mm) after heating between reference marks and L_O_ is the original length (mm) before heating.

To investigate the hypothesis air permeability through the prepared biocomposites, pore volume analysis expressed in terms of air flow rate was evaluated using a Bendtsen smoothness and porosity tester apparatus (N3500 model, Paper-testing association). The samples were measured for air permeability between the volumes of 5 mL/min and 3000 mL/min following ISO 5336-2013 standards, which is a non-destructive method to determine the air permeability of soft leather materials. The samples for analysis were cut in diameters of approximately 40 mm and the measurements were performed in triplicate and the average value recorded. According to the test, the amount of airflow through a given area of the sample at a constant pressure drop is measured. During the measurement, the sample is clamped over the air inlet of the Bendtsen apparatus using gaskets, and air is sucked through the sample via a pump. The air flow rate of per unit area at a given differential pressure was measured in mL/min.

### 2.5. Experimental Design

As the first stage of this study, various pre-trial experiments were performed to identify the most suitable protocol for the formulation of the biocomposite materials. The goal of this stage was to identify the most compatible mixture components and to successfully develop a biocomposite material with good properties. For this, Design of Experiments (DoEs) method was used, which is a widely applied technique to optimize experimental processes as well as to determine the optimal formulation of a specific mixture [35,36,37]. In this study, we used a mixture screening optimal design model, which consists of three types (D-, A- and I-optimal). Amongst all, the D-optimality was chosen considering this design model has proven to be one of most suitable factorial and screening designs used to identify significant variables. The details of the method can be found elsewhere [38,39,40].

In the present study, ten runs were selected by variation of the blending components in the mixtures and a 2-level factor D-optimal design was applied to evaluate the combined effects of 4 input components in weight percent (% w/w), which included BC, ML, PCL, and PLA. Based on pre-experimental trials, PVA was kept constant and the proportions of the prepared components in the mixtures were bound restricted within the limit feasible space of lower and upper constraints (Table 1).

The mixture components were assumed to be continuous variables and controllable by experiments with negligible errors. The responses selected were tensile strength and elastic modulus, coded as Y_1_ and Y_2_, respectively. The response function (Y) of the design model was partitioned into linear interactive components expressed using Equation (3).

(3) $${Y=\;\beta }_{1}X_{1}{\;+\;\beta }_{2}X_{2}{\;+\;\beta }_{3}X_{3}{\;+\;\beta }_{4}X_{4}+\;\varepsilon \;$$

Where, Y represents the response, β the variables coefficient, X is the input variable and ε is the error parameter.

### 2.6. Statistical Analysis

All measurements were recorded in triplicate and the results reported as the mean ± standard deviation. The design and analysis of the experiments were conducted with the statistical software of Design-Expert^®^ V11 (Stat-Ease Inc., Minneapolis, MN, USA) based on regression analysis of the experimental data. Analysis of Variance (ANOVA) was also applied to estimate the statistical parameters for which *p*-values < 0.05 were considered statistically significant.

## 3. Results and Discussion

### 3.1. Preparation of Biocomposite Leather

**Figure 1:**
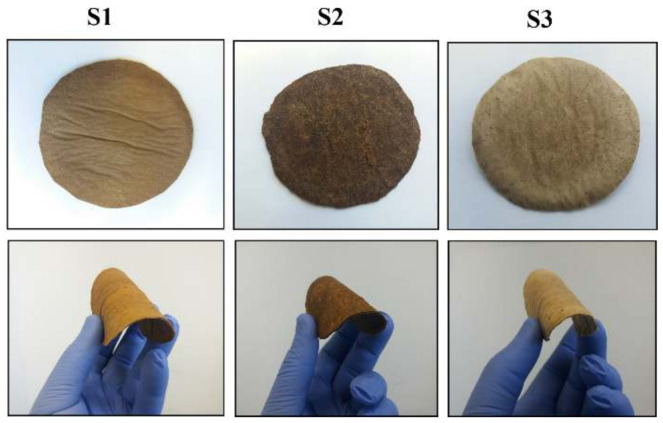
Appearance of prepared biocomposite leather-like materials (S1, S2 and S3).

Initially, three samples (S1, S2 and S3) of varying mixture components were prepared; they were of spherical shape and similar thickness within 1–2 mm. As can be seen from Figure 1, the samples were opaque brown, with smooth surface texture, considerable toughness and flexible.

### 3.2. FT-IR Analysis

**Figure 2:**
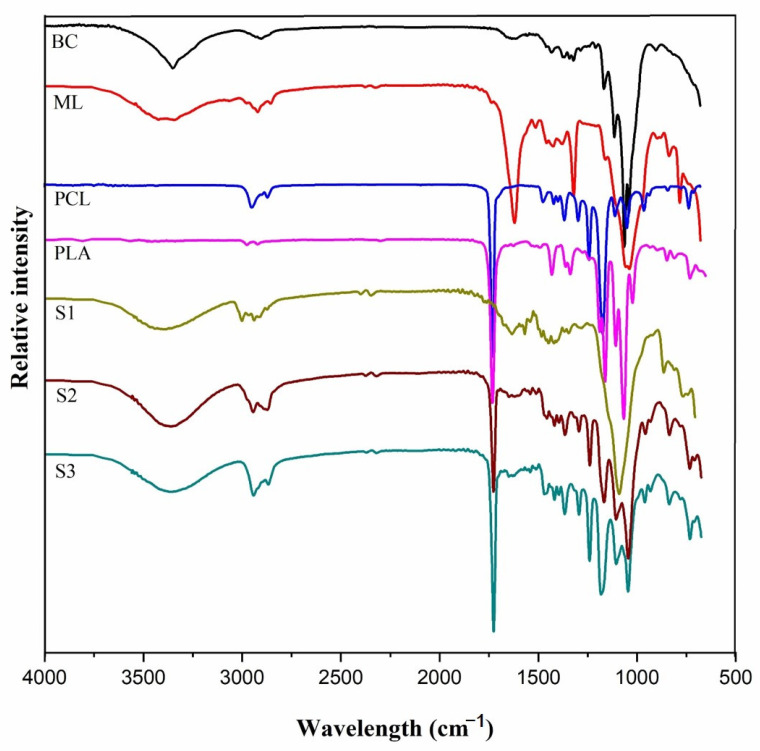
FTIR spectra bio-fillers (BC and ML), polymers matrix (PCL and PLA) and blended biocomposites (S1, S2, and S3).

Figure 2 is the FT-IR spectrum of bio-fillers, neat polymers and prepared blend biocomposites. The broad absorption peak between 3600–3200 cm^–1^ relates to O–H stretching vibrations from BC and ML [20,32]. The peaks at 2948 and 2860 cm^−1^ are ascribed to C–H stretching vibration of CH and CH_2_ from cellulose of the bio-fillers. The peaks were also present in neat PCL, PLA and the blend biocomposites. The strong peak at 1726 cm^–1^ is attributed to carbonyl (C=O) stretching that appeared for PCL, PLA and blended biocomposites. The peak did not appear for treated ML, which is because after alkali treatment hemicellulose was removed leading to the disappearance of C=O [41]. The band at 1615 cm^–1^ was attributed to the bending mode of absorbed water. The peaks at 1446 and 1326 cm^–1^ in the ML fiber represent the aromatic C–C ring stretching and C–H deformation in methyl, methylene and methoxy groups of lignocellulose [42]. It can be easily detected that all the bands corresponding to PCL and PLA matrix are present in all the biocomposites with the exception of hydroxyl bands of alcoholic and carboxylic groups intensity. Additionally, the C=O bending band at 1726 cm^–1^ appeared with significant intensity in S2 and S3, except for S1 since a multilayer assembly was form with the PCL layer between two bio-filler layers as subsequently confirmed by SEM. The peak at 1040 cm^–1^ was ascribed to C–O–C symmetric glycosidic stretching mode from carbohydrate components associated to cellulose [43].

### 3.3. Mechanical Tests

#### 3.3.1. Tensile Measurements

**Figure 3:**
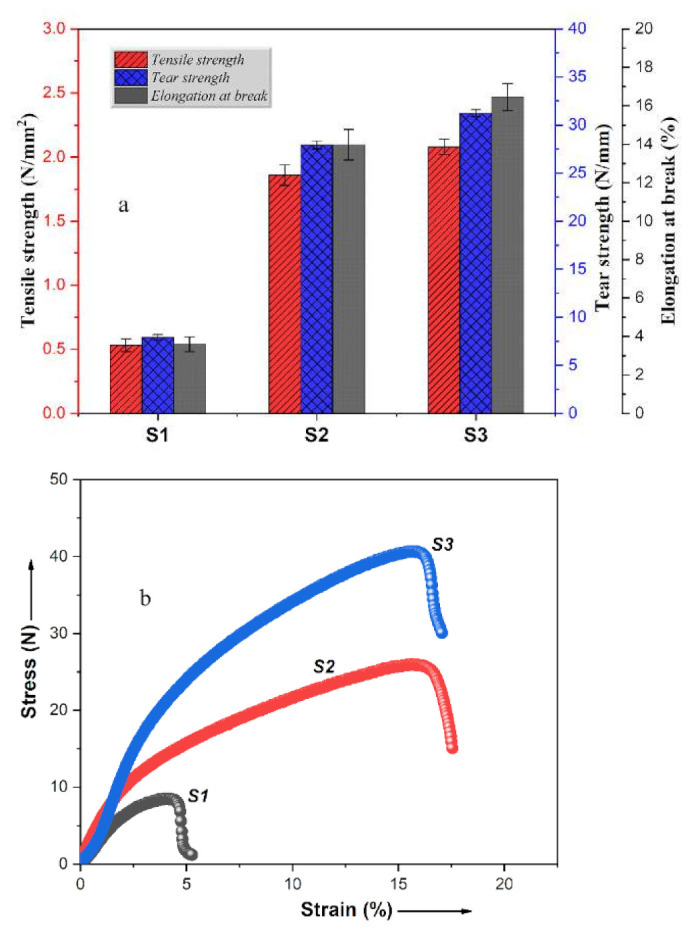
(**a**) Mechanical properties and (**b**) stress-strain plots of the prepared bio-leathers (samples S1, S2, S3).

In general, filler particle dispersion, interfacial adhesion and concentration of components are the main factors affecting a biocomposite’s mechanical properties [44]. In this work, the effect of bio-filler (BC and ML) and polymer matrix (PCL and PLA) on the mechanical properties of the biocomposites were investigated and compared. The mechanical properties including tensile strength, tear strength and elongation at break were tested for the three prepared biocomposite materials and the results are presented in Figure 3a. As can be seen, S1 has an average tensile strength of 0.53 ± 0.15 N/mm^2^. However, the direct blending of bio-fillers with PCL polymer matrix generated a better inter- and intra-particle interaction in the material leading to higher tensile strength of 1.86 ± 0.58 N/mm^2^ achieved for S2, which is 3 times higher than that of S1. This indicates that the blending of PCL with the treated bio-fillers of BC and ML created good interfacial adhesion between the particles and matrix. In addition, the low tensile strength of S1 may be attributed to the high concentration of ML that substantially affected the tensile strength. High loading incorporates rigid particles inside the matrix creating stress concentration zones that led to interfacial debonding under stress and probably premature sample disintegration [39]. By further modification of S2 with PLA to produce sample S3, a tensile strength of 2.08 ± 0.26 N/mm^2^ was obtained. The same trends as in the case of tensile strength can be observed for tear strength and elongation at break. The slight increase in mechanical properties of S3 could be attributed to the high mechanical properties of PLA. In addition, this may also be related to the different processing routes applied during the preparation process. However, the question why S3 shows better properties than S2, even though PLA is a brittle polymer may arise? According to literature studies, higher content of PLA in PLA/PCL mixtures significantly decreases the tensile strength and modulus of elasticity of the material as compared to the neat polymer. While incorporating PLA at low concentration into a PCL matrix improves the tensile properties of the material when compared to neat PCL matrix. At low concentration of PLA, the uniform dispersion of the polymers and effective interaction between the matrices is enhanced generating good mechanical properties [41,45].

Figure 3b shows the stress-strain curves of the prepared biocomposites in order to provide further insight into their tensile behaviors. According to results, it shows that the tensile modulus of S2 was increased by about 8.32% after the inclusion of 5 wt % PLA. This behavior can be attributed to an increase in strength of polymer matrix at the interfacial region due to the crystal nucleation effect of PLA on PCL. Similar results were obtained by Kakroodi et al. in a study on the preparation and characterization of PCL/PLA nanofibrillar composites [46].

#### 3.3.2. Dynamic Mechanical Analysis

**Figure 4:**
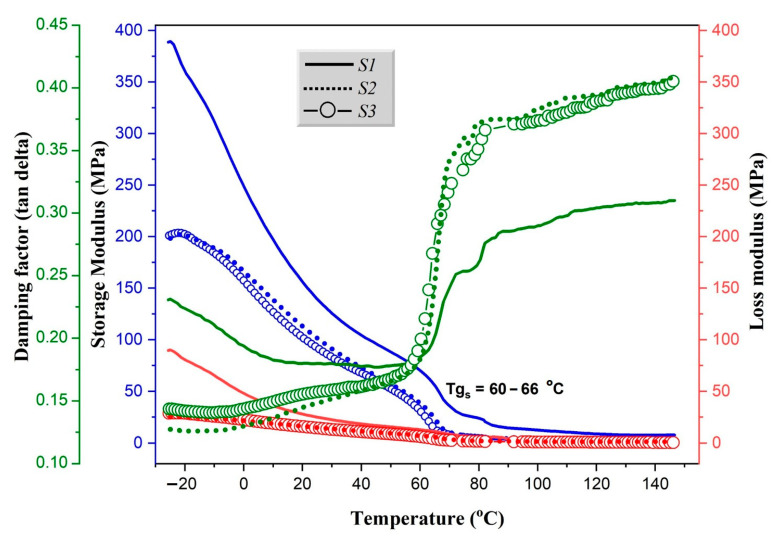
DMA of samples S1, S2 and S3–storage modulus, loss modulus and damping factor, with indicated T_g_ values.

Dynamic mechanical analysis (DMA) as a measure of the stiffness and damping properties of the material is reported in terms of storage modulus (E′), loss modulus (E″) and the damping factor (tan delta) of the material [41]. Figure 4 displays the behavior of the prepared biocomposites as a function of applied sinusoidal stress. According to obtained, several significant observations were deduced. Firstly, all prepared samples show similar viscoelastic behavior for tan delta, storage and loss moduli. However, the progressive decrease in storage modulus in the region from –25 °C to 60 °C was probably due to the increase weakening of inter- and intra-molecular bonds in the structural network, which affected the dynamic mechanical properties [47]. Further decrease in the storage modulus (> 60 °C) corresponds to the glass relaxation transitions of the samples, described as the T_g_ of the materials, where mechanical deformation starts to occur. As can be seen, the values of T_g_ of all the prepared materials were in the range of 60 to 66 °C. On the other hand, the damping factors of samples S2 and S3 were observed to be higher than that of S1, which can be attributed to the possible stiffening of polymers (PLA, PCL and PVA) and to a lesser extent cellulose chains in BC and ML [48,49]. The maximum average storage modulus of sample S1, S2 and S3 were determined as 110.30 ± 1.51, 78.34 ± 1.91 and 72.17 ± 1.60 MPa, respectively. The high value obtained for S1 compared to S2 and S3, indicates that the sample more stiff and rigid, which may be due to the high load of ML particles in the mixture. In order to better understand the thermo-mechanical features of the biocomposite materials, a control of the material behavior and optimal formulation for improved performance may be applicable. To introduce a temporal shape to the prepared biocomposites, it is expected that the material should be molded above its Tg and cooled to lower temperature [48]. Then the material will maintain its shape indefinitely until the Tg is exceeded, which may lead to disintegration.

### 3.4. Surface Morphology Analysis

**Figure 5:**
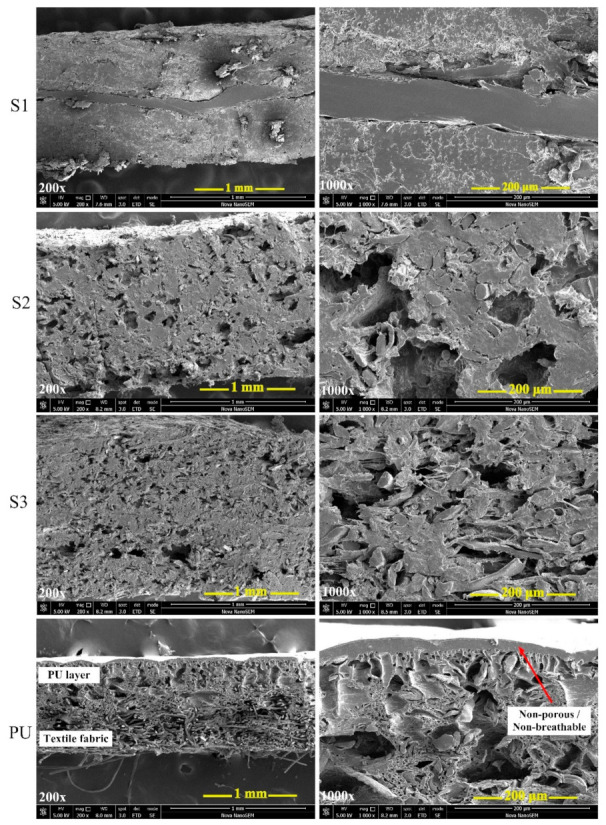
Cross section SEM images of the prepared biocomposite material (S1, S2 and S3) compared to commercial synthetic PU leather.

To evaluate the porosity and interfacial adhesion between the filler particulates and the polymer matrix, SEM analyses were performed for S1, S2, S3 and compared to commercial PU-based leather. The results are shown in Figure 5 and at first sight they seem to be similar. However, the surface and cross-section of the prepared biocomposite material (S1, S2 and S3) presents a compacted structure of nanofibers, indicating homogenous blending and interaction between bio-filler particles and the biodegradable polymer matrix, with randomly arranged pores throughout the biocomposite matrix to the surface. As observed, S1 showed a multilayer assembly as described in preparation. Overall, it is clear that bio-filler particles are randomly dispersed in the PCL/PLA matrix without particle agglomeration, indicating that good processing conditions were used in the blending and molding steps. The main difference between the biocomposites and synthetic PU leather can be seen on the surface. While the former shows a porous structure, PU material has a smooth thin non-porous layer on the surface, which shows non-breathability as compared to the prepared biocomposite with continuous pores to its surface that may allow air permeability and possibly breathable.

### 3.5. Optimization Study

#### 3.5.1. Experimental Design and ANOVA

The best composition formulation of the prepared biocomposite materials was reached with the help of Design-Expert^®^ V11 software. Table 2 shows the compositions of the experimental design for 10 runs in their coded forms determined by the model with the corresponding actual and predicted values. As can be seen, the experimental and the predicted values are within acceptable limits.

According to the Design Expert software, a linear Scheffe model well fitted the experimental data. In terms of the actual mixture composition, the interaction of the different biocomposite components in relation to tensile strength (Y_1_) and storage modulus (Y_2_) can be expressed mathematically as:

(4) $$Y_{1}\;=\;1{.20\;X}_{1}\;+\;0{.68\;X}_{2}\;+\;3{.39\;X}_{3}-0{.65\;X}_{4}\;$$

(5) $$Y_{2}\;=\;105{.02\;X}_{1}\;+\;195{.53\;X}_{2}+\;393{.14\;X}_{3}\;+\;240{.86X}_{4}\;$$

The fitness and significance of the model was assessed via ANOVA and the results are presented in Table 3. The deduced F-values for tensile strength and storage modulus indicated that the model is significant. In addition, *p*-values (Prob > F) less than 0.05 described the significance of the model terms. The factors X_1_, X_2_, and X_3_ proved to be significant model terms towards tensile strength and storage modulus responses. These observations were further supported by R^2^ and predicted R^2^ values, which were in close agreement with adjusted R^2^ (Table 3). This confirmed that the model could be used to navigate the formulation design space [50]. According to the design model equations, it is evident that not all the linear terms contributed positively in formulation with relation to the response output. The linear terms of BC, ML, and PCL showed the most significant positive effects on the responses of tensile strength and storage modulus. While PLA demonstrated less significant in terms of effects on responses.

#### 3.5.2. Optimization and Validation

Numerical optimization of the obtained valid linear model was applied for the different mixture compositions to determine the optimum response in relation to mechanical properties of the material. Table 4 shows the numerical optimization data of the predicted optimal formulation and their response values. To further validate the accuracy of the developed model, samples were prepared under predicted optimum formulation conditions and their response values re-measured. According to results obtained (Table 4), the experimental and predicted optimum values were reasonably close, which confirmed the validity and adequacy of the models. However, the optimized tensile strength (2.13 ± 0.29 N/mm^2^) and storage modulus (76.93 ± 1.63 MPa) were slightly higher. Further comparison between actual biocomposite sample, optimized and commercial PU-based leather material as presented in Table 4, shows that PU leather is still superior in mechanical integrity. However, comparing the obtained results to the technical report ISO/TR20879 as reference [49], the optimized biocomposite proved promising to be suitable for usage as shoe components in the footwear industry. Though the tensile and tear strength values were below the reference (normal footwear >10 N/mm^2^), elongation at break and elastic modulus presented values within the required reference range.

#### 3.5.3. Adhesion Analysis

**Figure 6:**
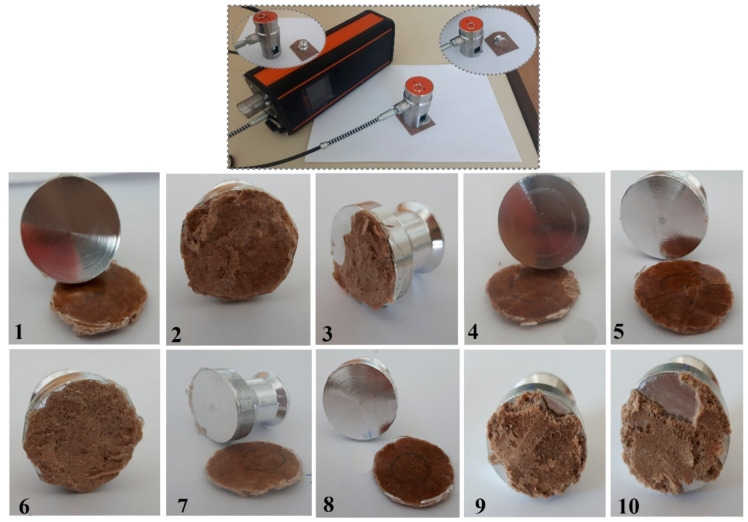
Pull-off test results of biocomposite samples (1–10) showing the different failures. Insert image depicting pull-off testometer with sample during testing.

Pull-off adhesion test on materials was performed to measure the adhesive strength of the given material with glue. The different kinds of failures of the prepared materials are shown in Figure 6. Further description of the failure behaviors for the different samples is provided in Table 5. As observed, samples 2, 3, 6, 9 and 10 partly depicted cohesive failure. This implies that the adhesive glue used was appropriate and the materials showed sufficiently good adhesion. On the other hand, samples 1, 4, 5, 7 and 8 demonstrated 100% glue failure. These tests conducted shows that the prepared biocomposite materials’ surface possess good adhesion properties, with the highest adhesion achieved for samples 1 and 6 as 7.28 ± 0.40 and 7.88 ± 0.49 MPa, respectively.

#### 3.5.4. Shape Stability, Water Absorption, Contact Angle and Pore Volume Analysis

In order to further specify the physico-chemical features of the prepared biocomposites, the apparent density, shape stability, water absorption, surface wettability and pore volume of the samples were evaluated. The apparent densities of the prepared materials were calculated to be in the range of (7.29–8.66) × 10^–4^ g/mm^3^. The shape stability of the prepared biocomposites as another significant property was investigated as a function of temperature. According to deduced results, the shape stability of the materials varied for different temperature treatments. Samples heated to 150 °C increased in dimensions (the length between reference points) from 1.6 to 2.8%. For temperature at 100 °C, the change decreased (0.6–1.34% increase), and for 50 °C no measurable change in length was observed. This demonstrates that for the investigated temperature range, the prepared biocomposites were to some extent stable at high temperatures.

Furthermore, Table 6 depicts the water absorption of biocomposites over a period of 24 h. Following the results obtained, the maximum water absorption capacity varied for the different samples in the range 35–60%. The absorbed water may be attributed to the polar functionality of the materials leading to polar-polar interaction between the free hydroxyl groups of the biocomposite matrix and water molecules. In addition, water absorption may have been contributed by the porous structure of the materials’ matrix. Overall, the absorption capacities of all investigated samples were in close agreement with quality requirements of upper shoe sole following BASF and German leather industry standards [34]. To further evaluate the water absorption properties of the prepared biocomposite, surface wettability was performed by determining the contact angle of water drops on the surface of the materials. According to results presented in Table 6, the contact angles of the samples was determined in the range 60–85°, which indicates the materials were all hydrophilic. Typically, contact angle values less than 90° correspond to high wettability. This was expected since the treated bio-fillers in the biocomposites were hydrophilic in nature that allows ease interaction with water molecules. A further plausible explanation for this is correlated to the alkali treatments of the bio-filler particles. Alkali treatment as a chemical method removes some non-cellulosic components (lignins and hemicelluloses) thereby increasing the accessibility of the free hydroxyl groups to interact hydrogen bonding between the fillers and the water molecules, thus increasing the hydrophilic nature of the biocomposites [51,52].

To investigate the hypothesis of the presence of free volume in the prepared biocomposites, pore volume analysis expressed in terms of air flow rate was evaluated for all the samples and the results presented in Table 6. This parameter demonstrates the extent of breathability of the materials. The biocomposite samples in this case showed high pore volume rates between 1000 to 3000 mL/min, which is supported by the porous matrix observed from the surface morphology analysis presented in SEM above. This indicates that the formulated biocomposite materials in the present study may possess breathable properties. Compared to the results obtained for synthetic polyurethane-based leather (as control) for which the pore volume rate was determined as zero, describes the PU material as non-breathable due to its non-porous surface layer. Overall, correlating the water absorption, surface wettability and pore volume data, shows that the performance of the biocomposite material relates to the polar functionality and surface morphology, which are vital features to be considered for improvement during future preparations.

## 4. Conclusions and Research Prospects

The purpose of the present study was to develop novel nonwoven fibrous biocomposite materials from readily available renewable biomass sources of microbial cellulose and maple tree leaves and biodegradable polymers. The biocomposite preparation was successfully optimized via DoE and according to the design model, the components BC, ML, and PCL were determined to have the most significant effect on the materials’ properties. Tensile and DMA analyses demonstrated the materials to be flexible, with considerable mechanical strength. The optimum tensile strength and storage modulus of prepared samples were determined as 2.13 ± 0.29 N/mm^2^ and 76.93 ± 1.63 MPa, respectively. SEM and pore volume analyses depicted the biocomposites to be porous and possibly breathable. Surface wettability demonstrated the biocomposites to be water loving with contact angles in the range of 60 to 85 °. In addition, the prepared biocomposites demonstrated good shape stability and adhesion properties. Overall, the obtained results show that the mechanical features of the prepared biocomposites are well suited for prospective application as leather substitute components for footwear. However, compared to conventional synthetic PU leather, the biocomposite will require further tailoring to meet the expected broader range of use. Yet at present, the prepared bio-leather shows a great potential to be used for undemanding footwear components.

Prospective research work will be focused on the sourcing of cheaper sustainable alternatives that will greatly contribute to the final performance and economic feasibility of the prepared bio-leathers. Considering the cost of some raw materials such as PLA and PCL still limit their applicability at a large scale, the present biomass-sourced nonwoven biocomposites can serve as suitable alternatives to some footwear components while reducing the rising negative environmental effects of the footwear industry.

## Figures and Tables

**Table 1:** Experimental ranges and levels of input variables and constraints of component proportions used for the optimal formulation of eco-leather biocomposites.

Component	Name	Low Limit (% w/w) (Coding Level = –1)	High Limit (% w/w) (Coding Level = +1)	Proportion Constraints (% w/w)
X_1_	BC	30	40	30 ≤ X_1_ ≤ 40
X_2_	ML	5	10	5 ≤ X_2_ ≤ 10
X_3_	PCL	15	20	15 ≤ X_3_ ≤ 20
X_4_	PLA	5	10	5 ≤ X_4_ ≤ 10

**Table 2:** D-optimal mixture design of biocomposite material formulation for actual and predicted values of tensile strength and elastic modulus.

Run	Individual Components				Response 1		Response 2	
	BC X_1_	ML X_2_	PCL X_3_	PLA X_4_	Tensile Strength (N/mm^2^)		Elastic Modulus (MPa)	
					Actual	Predicted	Actual	Predicted
1	+1	–1	+1	+1	2.10	1.96	146.10	143.69
2	–1	+1	+1	+1	1.80	1.77	152.30	136.52
3	–1	+1	–1	–1	1.68	1.61	49.53	43.61
4	+1	+1	–1	+1	0.88	0.59	84.20	98.24
5	–1	+1	+1	+1	0.89	0.96	159.20	146.26
6	+1	–1	–1	+1	1.27	1.40	103.30	88.39
7	–1	–1	+1	+1	1.72	1.75	73.98	99.19
8	+1	+1	+1	–1	2.00	2.09	137.30	151.11
9	+1	+1	–1	+1	0.46	0.68	76.48	83.27
10	+1	–1	+1	–1	1.36	1.34	79.20	71.31

**Table 3:** Analysis of variance (ANOVA) for design model.

Tensile Strength						
Source	Sum of SQUARES	df	Mean Square	F-Value	*p*-Value	
Model	4.86	6	0.8105	27.84	< 0.0001	Significant
Linear Mixtures	4.86	6	0.8105	27.84	< 0.0001	Significant
Lack of Fit	0.3785	3	0.1262			
R^2^	0.928					
Adjusted R^2^	0.895					
Predicted R^2^	0.846					
Std. Dev.	0.171					
**Storage modulus**						
Model	24,073.60	6	4012.27	14.40	< 0.0001	Significant
Linear Mixtures	24,073.60	6	4012.27	14.40	< 0.0001	Significant
Residual	3621.84	13	278.60			
Lack of Fit	3621.84	3	1207.28			
R^2^	0.869					
Adjusted R^2^	0.809					
Predicted R^2^	0.709					
Std. Dev.	16.69					

**Table 4:** Results of numerical optimization and validation tests.

Parameters	Goal	Experimental Region		Results				Model Desirability
		Lower	Upper	Optimized		Validation		
X_1_: BC	In range	30	40	37		37		0.901
X_2_: ML	In range	5	10	5		5		
X_3_: PCL	In range	15	20	18		18		
X_4_: PLA	In range	5	10	5		5		
Y_1_: Tensile strength	Maximized	0.46	2.10	1.94 ± 0.17		2.03 ± 0.29		
Y_2_: Storage modulus	Minimized	49.53	159.20	61.41 ± 1.69		63.93 ± 1.63		
**Samples**	**Responses**							
	Tensile strength (N/mm^2^)	Elongation at break (%)			Tear strength (N/mm)		Elastic modulus (MPa)	
Experimental	2.08 ± 0.06	16.45 ± 0.70			31.24 ± 0.35		72.17 ± 0.94	
Optimized	2.13 ± 0.29	19.23 ± 1.09			32.93 ± 1.33		76.93 ± 1.63	
PU Synthetic	5.28 ± 0.69	31.54 ± 2.32			79.20 ± 2.45		106.10 ± 2.70	

**Table 5:** Test results of adhesion and the type of failure for different samples.

Experimental Runs	Adhesion Force (MPa)	Type of Failure
1	7.28 ± 0.40	100% glue failure
2	6.37 ± 0.15	Partly cohesive failure
3	6.42 ± 0.23	Partly cohesive failure
4	7.05 ± 0.33	100% glue failure
5	4.56 ± 0.21	100% glue failure
6	7.88 ± 0.49	Partly cohesive failure
7	6.35 ± 0.35	100% glue failure
8	4.71 ± 0.20	100% glue failure
9	4.86 ± 0.24	Partly cohesive failure
10	4.55 ± 0.22	Partly cohesive failure

Cohesive failure: the same coating is on the surface and on the dolly face. Glue failure: When no coating/glue is present on the dolly, it records as a failure of the glue.

**Table 6:** Water absorption, contact angles and pore volume of prepared biocomposites materials.

Experimental Run	Water Absorption Capacity (%)	Water Contact Angle (°) θ_dH2O_	Pore Volume (mL/min)
1	44.89 ± 2.24	65.40 ± 1.99	2192 ± 109.60
2	39.37 ± 2.46	78.30 ± 1.06	1043 ± 47.14
3	37.63 ± 1.88	82.80 ± 1.87	1440 ± 72.50
4	46.05 ± 2.60	76.80 ± 2.09	1599 ± 79.95
5	45.17 ± 2.44	80.30 ± 1.52	1670 ± 83.50
6	52.50 ± 1.63	81.50 ± 2.68	2935 ± 146.75
7	45.71 ± 2.30	80.20 ± 1.96	1723 ± 86.15
8	46.17 ± 1.46	68.50 ± 1.07	1886 ± 94.30
9	54.37 ± 2.70	77.40 ± 0.88	3000 ± 150.33
10	47.17 ± 2.55	69.55 ± 1.23	2043 ± 102.15

## References

[1] Mittelstaedt J.D., Shultz C.J., Kilbourne W.E., Peterson M. (2014). Sustainability as Megatrend: Two Schools of Macromarketing Thought. J. Macromark..

[2] Varadarajan R. (2010). Strategic marketing and marketing strategy: Domain, definition, fundamental issues and foundational premises. J. Acad. Mark. Sci..

[3] Dixit S., Yadav A., Dwivedi P.D., Das M. (2015). Toxic hazards of leather industry and technologies to combat threat: A review. J. Clean. Prod..

[4] Nygren O., Wahlberg J.E. (1998). Speciation of chromium in tanned leather gloves and relapse of chromium allergy from tanned leather samples. Analyst.

[5] Nam C., Lee Y.A. (2019). Multilayered Cellulosic Material as a Leather Alternative in the Footwear Industry. Cloth. Text. Res. J..

[6] McNeill L., Moore R. (2015). Sustainable fashion consumption and the fast fashion conundrum: Fashionable consumers and attitudes to sustainability in clothing choice. Int. J. Consum. Stud..

[7] Lundblad L., Davies I.A. (2016). The values and motivations behind sustainable fashion consumption. J. Consum. Behav..

[8] Rathinamoorthy R., Muthu S.S. (2019). Consumer’s Awareness on Sustainable Fashion. Sustainable Fashion: Consumer Awareness and Education.

[9] Debnath S., Muthu S.S., Gardetti M.A. (2016). Natural Fibres for Sustainable Development in Fashion Industry. Sustainable Fibres for Fashion Industry: Volume 1.

[10] Kanagaraj J., Senthilvelan T., Panda R., Kavitha S. (2015). Eco-friendly waste management strategies for greener environment towards sustainable development in leather industry: A comprehensive review. J. Clean. Prod..

[11] Zhang Q., Khan M.U., Lin X., Yi W., Lei H. (2020). Green-composites produced from waste residue in pulp and paper industry: A sustainable way to manage industrial wastes. J. Clean. Prod..

[12] Samanta K.K., Basak S., Chattopadhyay S.K., Muthu S.S. (2015). Recycled Fibrous and Nonfibrous Biomass for Value-Added Textile and Nontextile Applications. Environmental Implications of Recycling and Recycled Products.

[13] Nourbakhsh A., Ashori A. (2010). Wood plastic composites from agro-waste materials: Analysis of mechanical properties. Bioresour. Technol..

[14] Vaisanen T., Haapala A., Lappalainen R., Tomppo L. (2016). Utilization of agricultural and forest industry waste and residues in natural fiber-polymer composites: A review. Waste Manag..

[15] Schettini E., Santagata G., Malinconico M., Immirzi B., Scarascia Mugnozza G., Vox G. (2013). Recycled wastes of tomato and hemp fibres for biodegradable pots: Physico-chemical characterization and field performance. Resour. Conserv. Recycl..

[16] Shahid-ul-Islam S.M., Mohammad F. (2013). Perspectives for natural product based agents derived from industrial plants in textile applications—A review. J. Clean. Prod..

[17] Sen T., Reddy H.J. (2011). Various industrial applications of hemp, kinaf, flax and ramie natural fibres. Int. J. Innov. Manag. Technol..

[18] Cao H., Wool R.P., Bonanno P., Dan Q., Kramer J., Lipschitz S. (2014). Development and evaluation of apparel and footwear made from renewable bio-based materials. Int. J. Fash. Des. Technol. Educ..

[19] Ngwabebhoh F.A., Yildiz U. (2019). Nature-derived fibrous nanomaterial toward biomedicine and environmental remediation: Today’s state and future prospects. J. Appl. Polym. Sci..

[20] Alemdar A., Sain M. (2008). Isolation and characterization of nanofibers from agricultural residues–Wheat straw and soy hulls. Bioresour. Technol..

[21] Iguchi M., Yamanaka S., Budhiono A.J. (2000). Bacterial cellulose—A masterpiece of nature’s arts. J. Mater. Sci..

[22] Gallegos A.M.A., Carrera S.H., Parra R., Keshavarz T., Iqbal H.M. (2016). Bacterial cellulose: A sustainable source to develop value-added products—A review. BioResources.

[23] Karim Z., Afrin S., Jawaid M., Boufi S., Abdul Khalil H.P.S. (2017). Bacterial cellulose: Preparation and characterization. Cellulose-Reinforced Nanofibre Composites.

[24] Shah N., Ul-Islam M., Khattak W.A., Park J.K. (2013). Overview of bacterial cellulose composites: A multipurpose advanced material. Carbohydr. Polym..

[25] García C., Prieto M.A. (2018). Bacterial cellulose as a potential bioleather substitute for the footwear industry. Microb. Biotechnol..

[26] Eluxe Magazine Eco-Friendly Vegan Leathers. https://eluxemagazine.com/fashion/5-truly-eco-friendly-vegan-leathers/.

[27] Material District Malai Biocomposite. https://materialdistrict.com/material/malai/.

[28] Politis S.N., Colombo P., Colombo G., Rekkas D.M. (2017). Design of experiments (DoE) in pharmaceutical development. Drug Dev. Ind. Pharm..

[29] Paulo F., Santos L. (2017). Design of experiments for microencapsulation applications: A review. Mater. Sci. Eng. C.

[30] Tepe O., Dursun A.Y. (2014). Exo-pectinase production by Bacillus pumilus using different agricultural wastes and optimizing of medium components using response surface methodology. Environ. Sci. Pollut. Res..

[31] Durakovic B. (2017). Design of experiments application, concepts, examples: State of the art. Period. Eng. Nat. Sci..

[32] Bandyopadhyay S., Saha N., Brodnjak U.V., Saha P. (2018). Bacterial cellulose based greener packaging material: A bioadhesive polymeric film. Mater. Res. Express.

[33] Chen H., Yu Y., Zhong T., Wu Y., Li Y., Wu Z., Fei B. (2017). Effect of alkali treatment on microstructure and mechanical properties of individual bamboo fibers. Cellulose.

[34] BASF (2007). Pocket Book for Leather Technologist.

[35] Yin H., Chen Z., Gu Z., Han Y. (2009). Optimization of natural fermentative medium for selenium-enriched yeast by d-optimal mixture design. LWT Food Sci. Technol..

[36] Jeirani Z., Jan B.M., Si Ali B., Noor I.M., Chun Hwa S., Saphanuchart W. (2012). The optimal mixture design of experiments: Alternative method in optimizing the aqueous phase composition of a microemulsion. Chemom. Intell. Lab. Syst..

[37] Mura P., Furlanetto S., Cirri M., Maestrelli F., Marras A.M., Pinzauti S. (2005). Optimization of glibenclamide tablet composition through the combined use of differential scanning calorimetry and d-optimal mixture experimental design. J. Pharm. Biomed. Anal..

[38] Myers R.H., Montgomery D.C., Anderson-Cook C.M. (2016). Response Surface Methodology: Process and Product Optimization Using Designed Experiments.

[39] Prithivirajan R., Jayabal S., Bharathiraja G. (2015). Bio-based composites from waste agricultural residues: Mechanical and morphological properties. Cellul. Chem. Technol..

[40] Quiles-Carrillo L., Montanes N., Pineiro F., Jorda-Vilaplana A., Torres-Giner S. (2018). Ductility and Toughness Improvement of Injection-Molded Compostable Pieces of Polylactide by Melt Blending with Poly(ε-caprolactone) and Thermoplastic Starch. Materials.

[41] Akos N.I., Wahit M.U., Mohamed R., Yussuf A.A. (2013). Preparation, characterization, and mechanical properties of poly (ε-caprolactone)/polylactic acid blend composites. Polym. Compos..

[42] Reddy K.O., Uma Maheswari C., Muzenda E., Shukla M., Rajulu A.V. (2016). Extraction and characterization of cellulose from pretreated ficus (peepal tree) leaf fibers. J. Nat. Fibers.

[43] Laaziz S.A., Raji M., Hilali E., Essabir H., Rodrigue D., Bouhfid R. (2017). Bio-composites based on polylactic acid and argan nut shell: Production and properties. Int. J. Biol. Macromol..

[44] Saba N., Mohammad F., Pervaiz M., Jawaid M., Alothman O.Y., Sain M. (2017). Mechanical: Morphological and structural properties of cellulose nanofibers reinforced epoxy composites. Int. J. Biol. Macromol..

[45] Saba N., Safwan A., Sanyang M.L., Mohammad F., Pervaiz M., Jawaid M., Alothman O.Y., Sain M. (2017). Thermal and dynamic mechanical properties of cellulose nanofibers reinforced epoxy composites. Int. J. Biol. Macromol..

[46] Kakroodi A.R., Kazemi Y., Rodrigue D., Park C.B. (2018). Facile production of biodegradable PCL/PLA in situ nanofibrillar composites with unprecedented compatibility between the blend components. Chem. Eng. J..

[47] Jeyapalina S., Attenburrow G.E., Covington A.D. (2007). Dynamic mechanical thermal analysis (DMTA) of leather part 1: Effect of tanning agent on the glass transition temperature of collagen. J. Soc. Leather Technol. Chem..

[48] Misra M., Pandey J.K., Mohanty A. (2015). Biocomposites: Design and Mechanical Performance.

[49] ISO (2007). ISO/TR 20879:2007. Footwear—Performance Requirements for Components for Footwear—Uppers.

[50] Cornell J.A. (2011). Experiments with Mixtures: Designs, Models, and the Analysis of Mixture Data.

[51] Nguyen M.H., Kim B.S., Ha J.R., Song J. (2011). Effect of plasma and NaOH treatment for rice Husk/PP composites. Adv. Compos. Mater..

[52] Essabir H., Ouadi Bensalah M., Rodrigue D., Bouhfid R., Qaiss A. (2016). Biocomposites based on Argan nut shell and a polymer matrix: Effect of filler content and coupling agent. Carbaohydrate Polym..

